# Impact of postoperative baseline MRI on diagnostic confidence and performance in detecting local recurrence of soft-tissue sarcoma of the limb

**DOI:** 10.1007/s00256-023-04341-4

**Published:** 2023-05-02

**Authors:** Sophia Samira Goller, Paul Reidler, Jan Rudolph, Johannes Rückel, Nina Hesse, Vanessa Franziska Schmidt, Hans Roland Dürr, Alexander Klein, Lars Hartwin Lindner, Dorit Di Gioia, Isabella Kuhn, Jens Ricke, Bernd Erber

**Affiliations:** 1 Department of Radiology, University Hospital, LMU Munich, Munich, Germany; 2 Institute of Neuroradiology, University Hospital, LMU Munich, Munich, Germany; 3 Department of Orthopaedics and Trauma Surgery, Musculoskeletal University Center Munich (MUM), University Hospital, LMU Munich, Munich, Germany; 4 Department of Medicine III, University Hospital, LMU Munich, Munich, Germany

**Keywords:** Soft-tissue sarcoma, Local recurrence, Magnetic resonance imaging, Postoperative baseline MRI

## Abstract

**Objective:**

To evaluate the impact of a postoperative baseline (PB) MRI on diagnostic confidence and performance in detecting local recurrence (LR) of soft-tissue sarcoma (STS) of the limb.

**Materials and methods:**

A total of 72 patients (8 with LR, 64 without LR) with primary STS of the limb were included. Routine follow-up MRI (1.5 T) at 6 and approximately 36 months (mean_LR_: 39.7 months; mean_no LR_: 34.9 months) after multimodal therapy or at time of LR were assessed by three independent readers using a 5-point Likert scale. Furthermore, the following imaging parameters were evaluated: presence of a mass, signal characteristics at T2- and T1-weighted imaging, contrast enhancement (CE), and in some of the cases signal intensity on the apparent diffusion coefficient (ADC). U-test, McNemar test, and ROC-analysis were applied. Interobserver reliability was calculated using Fleiss kappa statistics. A *p* value of 0.05 was considered statistically significant.

**Results:**

The presence of a PB MRI significantly improved diagnostic confidence in detecting LR of STS (*p* < 0.001) and slightly increased specificity (mean specificity without PE 74.1% and with presence of PB MRI 81.2%); however, not to a significant level. The presence of a mass showed highest diagnostic performance and highest interreader agreement (AUC [%]; *κ*: 73.1–83.6; 0.34) followed by T2-hyperintensity (50.8–66.7; 0.08), CE (52.4–62.5; 0.13), and T1-hypointensity (54.7–77.3; 0.23). ADC showed an AUC of 65.6–96.6% and a *κ* of 0.55.

**Conclusion:**

The presence of a PB MRI increases diagnostic confidence in detecting LR of STS of the limb.

## Introduction

Soft-tissue sarcoma (STS) is a rare entity, accounting for approximately 1% of malignancies in adulthood [1, 2]. STS arises from undifferentiated mesenchymal stem cells and occurs in different parts of the body [1, 2] with liposarcoma being the most common histological subtype in the limb accounting for approximately 23% [3].

Diagnosis, treatment, and follow-up are based on a multidisciplinary approach. The initial tumor stage, which is commonly assessed according to the current Union for International Cancer Control stage classification system, is crucial for both therapy and prognosis [4]. While surgery is the mainstay of treatment, local and systemic therapies including chemotherapy (ChT), radiation therapy (RT), and regional hyperthermia (RHT) are additionally applied to surgery for deep-seated STS and tumor sizes of more than 5 cm to prevent local recurrence (LR) and metastatic spread [1, 5–9]. Further, the risk of LR depends on factors like histological tumor entity or multifocally positive margins after surgery [10]. Approximately 16% of patients who have been treated for STS develop LR after a follow-up period of 24 months [9].

While there is no clear general consent in the literature, routine follow-up in multimodally treated high-grade STS is recommended every 3 to 4 months during the first 3 years by ESMO guidelines and every 3 to 6 months by NCCN guidelines; while in the later course follow-up is recommended every 6 months during the fourth and fifth year, and once a year later on [4, 11–13]. While CT is considered the imaging modality of choice to screen for lung metastases, MRI is used for the detection of LR following the recommendations of the American College of Radiology Appropriateness Criteria guidelines [14].

However, the impact of postoperative baseline (PB) and follow-up MRI in multimodally treated STS is not completely clear as diagnostic difficulties due to post-therapeutic alterations like changes in anatomy, muscular and subcutaneous edema as well as scar tissue in the treated area may severely impair the detection rate of LR [15–17]. In particular, it is still unclear whether the availability of a PB MRI scan after primary treatment may potentially increase the diagnostic performance of later follow-up MRI scans by improving the discrimination between post-treatment changes and locally recurrent STS [18].

Therefore, this study aimed to investigate the benefit of a PB MRI on the diagnostic performance and confidence in detecting LR of STS of the limb. Furthermore, we qualitatively evaluated different standard MRI parameters over time.

## Materials and methods

### Subjects

Approval from the Institutional Review Board was obtained (No. 17–279), and in keeping with the policies for a retrospective study design, informed consent was not required.

A total of 72 consecutive individuals (over the age of 18 years) who underwent multimodal therapy including surgery and optionally a combination of ChT, RT, and RHT for the diagnosis of high-grade STS of the limb (grade 2 and 3) between January 2015 and December 2021 were included. Tumor grading was confirmed histopathologically. Patients’ characteristics are summarized in Table 1. Patients were retrospectively identified via a full-text report query within the local radiology information system (RIS)/picture archiving and communication system (PACS) (Siemens Healthineers, Erlangen, Germany) using the search term “sarcoma” (initially 537 reports) and subsequent specification to “soft-tissue sarcoma.” The results were further filtered concerning the availability of follow-up MRI scans at 6 and approximately 36 months after multimodal therapy or at time of LR, respectively.

**Table 1:** Patients’ characteristics

	No LR	LR
Total	64	8
Age		
Mean	63	61
Histopathological subtype		
Synovial sarcoma	11	2
Pleomorphic sarcoma	26	5
Leiomyosarcoma	8	
Liposarcoma	6	1
Myxofibrosarcoma	3	
Malignant peripheral nerve sheath tumor	3	
NOS sarcoma	2	
Extraskeletal chondrosarcoma	3	
Epithelioid sarcoma	2	
Primary tumor site		
Thigh and waist	43	5
Lower leg	11	1
Forearm	4	1
Upper arm	6	1
Grading		
G2	34	4
G3	30	4

### Assessment of STS local recurrence

Standard of reference for the diagnosis of LR in STS was histology in all eight cases.

Standard of reference in patients without LR was a further 6-month follow-up without clinical (diagnosed by orthopedic tumor specialists) or MR-morphological signs (diagnosed by different musculoskeletal radiologists, others than the readers in this study) of tumor recurrence.

### MRI data acquisition

All MRI studies were performed on a 1.5 T unit (Magnetom Avanto, Siemens Healthineers, Erlangen, Germany). Precontrast sequences included coronal short-tau inversion recovery (STIR) sequences, axial and coronal T1-weighted, axial T2-weighted turbo spin-echo (TSE), axial proton density (PD)-weighted, and axial diffusion-weighted (DWI) echo-planar imaging (EPI) sequences. Postcontrast sequences consisted of axial and coronal T1-weighted fat saturated images.

### Image review and analysis

Image review and qualitative analysis were performed using a commercially available PACS (Nexus AG, Donaueschingen, Germany). MR studies were anonymized and independently reviewed by three radiologists with different experiences in musculoskeletal imaging, who were blinded to all clinical information. To avoid interpretation bias, cases with and without LR were pooled together and the reading order was randomly assigned.

Reader 1 was a board-certified musculoskeletal-specified radiologist with 7 years of experience, reader 2 a board-certified radiologist with 6 years of experience, and reader 3 a resident with 5 years of experience, respectively. In each case, images of the second MR examination were assessed without use of the PB MRI, and in a second session, 5 weeks later, with use of the 6-month PB MRI.

MRI findings were systematically classified on a 5-point Likert scale applying the following lesion classification: 1—definitely no LR; 2—probably no LR; 3—intermediate risk for LR—short term follow-up or biopsy needed; 4—probably LR; 5—definitely LR.

For calculation of readers’ confidence, Likert scale ratings were transferred as follows: 1—definite decision (Likert 1 and 5); 2—probable decision (Likert 2 and 4); 3—uncertain decision (Likert 3).

Likert scale ratings 1 and 2 were grouped and rated as the correct identification of no LR, and 3–5 were grouped and rated as the correct identification of LR or in need of clarification.

For evaluation of imaging characteristics, the following features were used according to previous literature [18]:

1.  Presence of a mass in all sequences (no minimum size defined).
2. Increased signal intensity in T2-weighted sequences (0 = no signal alteration; 1 = increased signal intensity compared to surrounding muscle) in the area of resection compared to healthy tissue.
3.  Reduced signal intensity in T1-weighted sequences (0 = no signal alteration; 1 = reduced signal intensity compared to surrounding muscle) in the area of resection compared to healthy tissue.
4.  Enhanced uptake of contrast in T1-weighted fat saturated images (0 = no signal alteration; 1 = increased contrast enhancement compared to surrounding muscle) in the area of resection compared to healthy tissue.
5.  Reduced signal intensity in ADC sequences (0 = no signal alteration; 1 = reduced signal intensity compared to surrounding muscle) in the area of resection compared to healthy tissue.

**Fig. 1:**
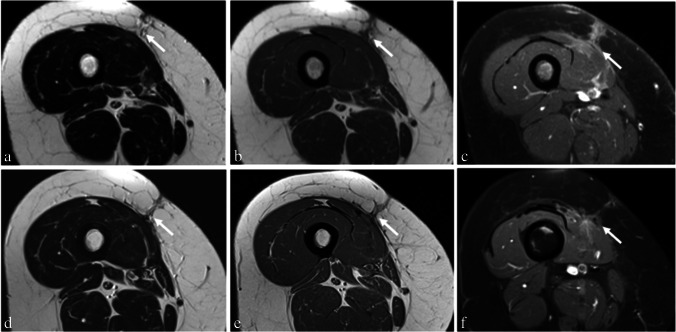
Patient example without LR in synovial sarcoma of the right thigh after multimodal therapy. Images of a 45-year-old female patient six (**a**–**c**) and 31 months (**d**–**f**) after multimodal therapy for a synovial sarcoma of the right thigh without signs of LR in further follow-up. **a** T2-weighted image shows slight diffuse increased signal intensity in the area of resection (arrow) while slight T1-hypointensity in subcutaneous fat was evaluated (**b**). Also, marked diffuse contrast uptake is shown in the area of resection (**c**). In a T2-weighted image 36 months after therapy (**d**), slight diffuse edema is present, while marked contrast uptake (arrow) in the area of resection is present (**f**). Minimal T1-hypointensity was detected in the area of resection (**e**)

**Fig. 2:**
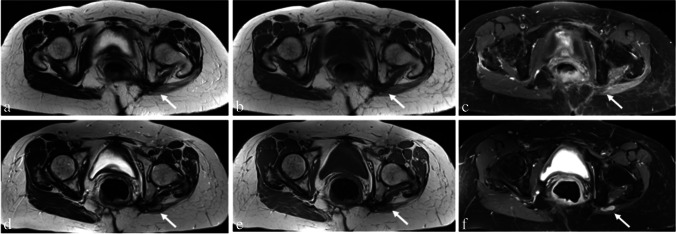
Patient example with LR in synovial sarcoma of the left hip after multimodal therapy. Images of a 62-year-old female patient six (**a**–**c**) and 37 months (**d**–**f**) after multimodal therapy for a synovial sarcoma of the left hip with histologically proven LR. **a** T2-weighted image shows slight diffuse increased signal intensity in the area of resection (arrow), while slight T1-hypointensity was evaluated (**b**). Also, slight diffuse contrast uptake is shown in the area of resection (**c**). Imaging 37 months after therapy revealed a mass. A nodular-shaped T2-hyperintensity (**d**), T1-hypointensity (**e**), and contrast uptake were evaluated (arrows) (**f**)

An example of patients with and without signs of LR are shown in Figs. 1 and 2.

### Statistical analysis

Statistical analysis was performed using R-Studio (Version 4.0.4, RStudio Inc., Boston, MA). Receiver operating characteristic (ROC) curves were plotted to analyze the diagnostic performance of all imaging characteristics and to calculate areas under the curve (AUC). Categorial variables of 5-point Likert scale were compared using U-test for dependent samples. Sensitivity, specificity, true positive, and negative rate were calculated for each reader and Fleiss kappa statistics were evaluated for interobserver reliability. The degree of agreement was classified using kappa values according to the recommendation by Landis and Koch [19] as follows: 0.00–0.20, slight agreement; 0.21–0.40, fair agreement; 0.41–0.60, moderate agreement; 0.61–0.80, substantial agreement; 0.81–1.00, almost-perfect agreement. The limit of statistical significance was set at 0.05, resulting in 95% confidence intervals (CIs).

## Results

### Patients

Out of the study cohort (*n* = 72), none of the patients had signs of LR in first MRI 6 months after multimodal therapy. In the second MRI, eight showed signs of LR based on the above-mentioned standard of reference. Mean age of patients with LR was 62.5 years, and 61.3 years for patients without LR. Mean time (min–max ± standard deviation (SD)) between surgery and detection of LR in MRI was 39.7 (15.4–107.2 ± 30.0) months with a median of 32.1 months, and between surgery and corresponding second MRI for patients without LR, a mean of 34.9 (16.4–49.7 ± 7.0) months with a median of 33.2 months.

Most common histological subtype in both groups was pleomorphic sarcoma (4/8 in LR patients and 23/64 in patients without LR), while the thigh was the most common tumor site.

### Improvement of diagnostic confidence with help of postoperative baseline MRI

Subjectively assessed diagnostic confidence in diagnosis of recurrence of STS was significantly better for all readers if a PB MRI was available at time of evaluation compared to an assessment without the availability of a PB MRI.

For reader 1, mean score improved from 1.9 (min 1.0; max 3.0; SD 0.8) at assessment without PB MRI to 1.6 (min 1.0; max 3.0; SD 0.7) at assessment with PB MRI (*p* < 0.001).

**Fig. 3:**
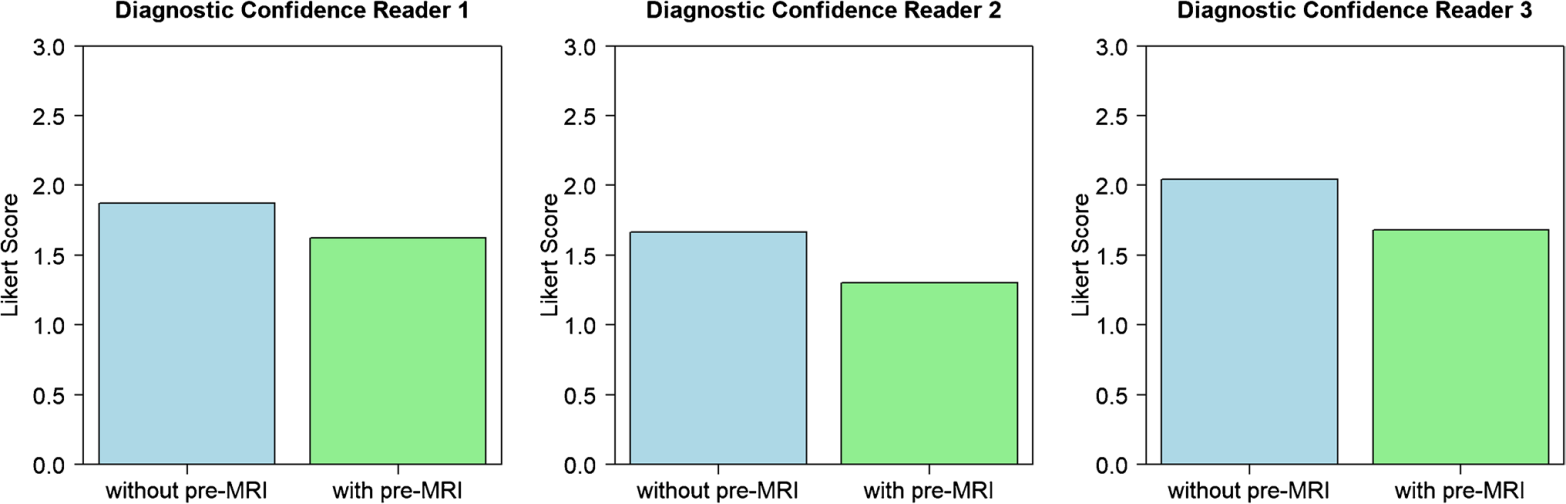
Diagnostic confidence. Barplots showing mean ratings of Likert scale for the readers for evaluation of MRI 36 months after multimodal therapy for STS without and with availability of a PB MRI. Differences were significant for all readers

For reader 2, mean score improved from 1.7 (min 1.0; max 3.0; SD 0.7) at assessment without PB MRI to 1.3 (min 1.0; max 3.0; SD 0.6) at assessment with PB MRI (*p* < 0.001), while for reader 3, mean score improved from 2.0 (min 1.0; max 3.0; SD 0.5) at assessment without PB MRI to 1.7 (min 1.0; max 3.0; SD 0.6) at assessment with PB MRI (*p* < 0.001). Ratings are shown in Fig. 3.

### Influence of postoperative baseline MRI on readers’ accuracy

Sensitivities of all readers in detecting LR were mostly constant between assessment without or with PB MRI. Reader 1 had a sensitivity of 75% (6/8) without PB MRI and of 75% (6/8) with PB MRI for identification of LR (correct identification of LR; Likert 4 and 5). Reader 2 had a sensitivity of 50% (4/8) without PB MRI and of 62.5% (5/8) with PB MRI, and reader 3 a sensitivity of 75% (6/8) without PB MRI and of 75% (6/8) with PB MRI.

Specificities in detection of LR (correct identification of no-LR; Likert 1 and 2) slightly increased for all readers between assessment without and with PB MRI; however, differences were not significant. Specificity was 66.1% for reader 1 (43/64) without PB MRI and 75.0% (48/64) with PB MRI (*p* = 0.23).

Specificity was 78.1% for reader 2 (50/64) without PB MRI and 85.9% (55/64) with PB MRI (*p* = 0.34).

And for reader 3, specificity was 78.1% (50/64) without PB MRI and 82.8% (53/64) with PB MRI (*p* = 0.37).

For reader 1, the true positive rate (and true negative rate) was 22.2% (95.5%) without PB MRI and 39.3% (96%) with PB MRI.

For reader 2, the true positive rate (and true negative rate) was 36.4% (92.6%) without PB MRI and 41.7% (94.7%) with PB MRI.

For reader 3, the true positive rate (and true negative rate) was 30.0% (96.1%) without PB MRI and 35.3% (96.4%) with PB MRI.

### Diagnostic performance of imaging characteristics

The presence of a mass showed highest diagnostic performance of all assessed imaging parameters in detection of LR of STS. For reader 1, it had an AUC of 83.6% (95% CI: 70.4–96.8%), for reader 2 an AUC of 73.1% (54.5–91.6%), and for reader 3 an AUC of 82.8% (69.5–96.1%). Differences between both groups of LR and of no LR regarding the presence of a mass were significant for all readers (*p* < 0.001).

AUCs for signal changes in T2-weighted imaging were 60.2% (41.2–79.1%) for reader 1, 66.7% (52.9–80.5%) for reader 2, and 50.8% (49.3–52.3%) for reader 3. Differences between both groups of LR and no LR regarding signal changes in T2-weighted imaging were not significant for all readers (*p* = 0.28; *p* = 0.08; *p* = 0.76).

AUCs for signal changes in T1-weighted imaging were 54.7% (38.0–71.3%) for reader 1, 59.7% (41.2–78.3%) for reader 2, and 77.3% (59.1–95.6%) for reader 3. Differences between both groups of LR and no LR regarding signal changes in T1-weighted imaging were only significant for reader 3 (*p* = 0.51; *p* = 0.21; *p* < 0.001).

AUCs for contrast enhancement were 56.2% (39.1–73.4%) for reader 1, 62.5% (45.2–79.8%) for reader 2, and 52.4% (49.7–55.0%) for reader 3. Differences between both groups of LR and no LR regarding contrast enhancement were not significant for all readers (*p* = 0.50; *p* = 0.19; *p* = 0.55).

AUCs for signal changes in ADC imaging were 65.6% (47.4–83.8%) for reader 1, 95.2% (88.8–100.0%) for reader 2, and 96.6% (91.9–100.0%) for reader 3. Differences between both groups of LR and no LR regarding ADC-signal changes were significant for all readers (*p* < 0.01; *p* < 0.01; *p* < 0.001).

**Fig. 4:**
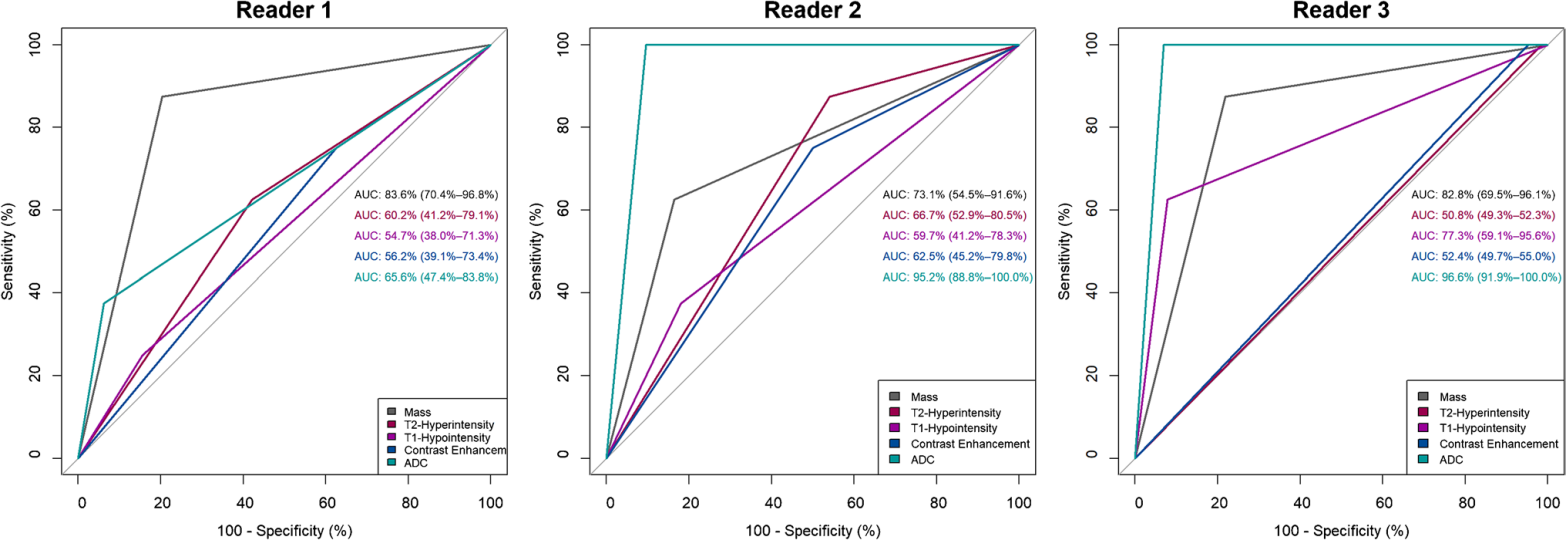
Diagnostic performance of imaging characteristics. AUCs with confidence intervals (in brackets) for all imaging characteristics for each reader: Presence of a mass in all sequences, increased signal intensity in T2-weighted sequences, reduced signal intensity in T1-weighted sequences, enhanced uptake of contrast in T1-weighted fat saturated images, and signal intensity in ADC sequences

ROC curves are displayed in Fig. 4.

### Progression of imaging characteristics

**Fig. 5:**
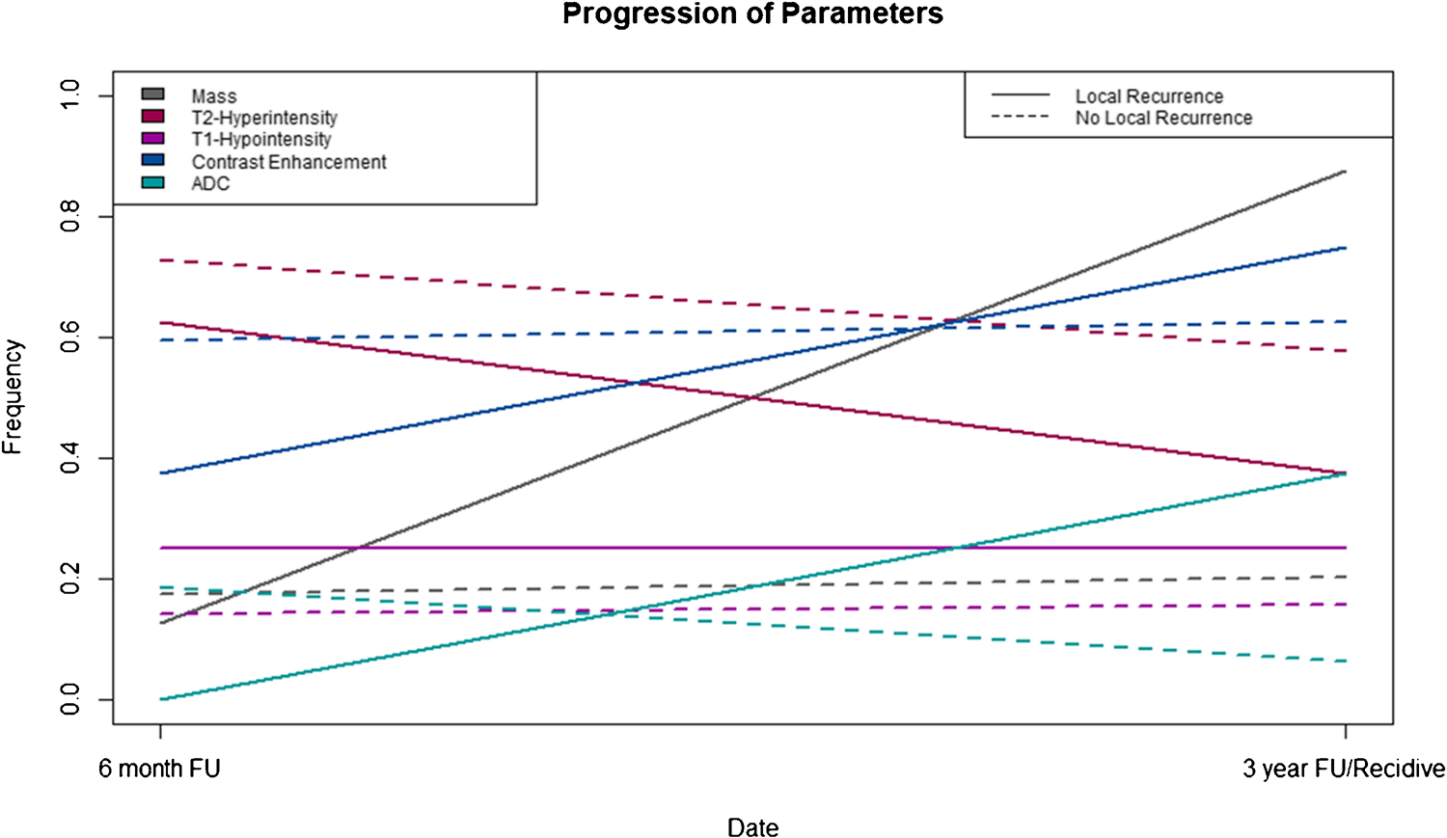
Progression of imaging characteristics. Line plots showing the frequency of the presence of each imaging sign at 6-month follow-up and at 36 months and the time of local recurrence of STS

Progression of imaging characteristics is shown in Fig. 5.

In the cohort without LR, most imaging characteristics remained predominantly constant between the PB MRI at 6 months after resection and follow-up examination approximately at 36 months after surgery:

For reader 1, the presence of a mass was positive in 17.2% (reader 2: 15.6%; reader 3: 21.9%) at PB MRI and 20.3% (reader 2: 15.6%; reader 3: 21.9%) at follow-up examination for cases without LR as well as in 12.5% (reader 2: 0%; reader 3: 37.5%) at PB MRI and 87.5% (reader 2: 62.5%; reader 3: 87.5%) at follow-up examination for cases with LR.

For reader 1, signal changes in T2-weighted images were rated positive in 71.9% (reader 2: 64.1%; reader 3: 100%) at PB MRI and 57.8% (reader 2: 51.6%; reader 3: 98.4%) at follow-up examination for cases without LR as well as in 62.5% (reader 2: 62.5%; reader 3: 100%) at PB MRI and 37.5% (reader 2: 87.5%; reader 3: 100%) at follow-up examination for cases with LR.

For reader 1, signal changes in T1-weighted images were rated positive in 14.1% (reader 2: 15.6%; reader 3: 9.4%) at PB MRI and 15.6% (reader 2: 17.1%; reader 3: 7.8%) at follow-up examination for cases without LR as well as in 25.0% (reader 2: 0%; reader 3: 12.5%) at PB MRI and 25.0% (reader 2: 37.5%; reader 3: 62.5%) at follow-up examination for cases with LR.

For reader 1, contrast enhancement was rated positive in 59.4% (reader 2: 62.5%; reader 3: 96.9%) at PE and 62.5% (reader 2: 46.9%; reader 3: 93.8%) at follow-up examination for cases without LR as well as in 37.5% (reader 2: 50.0%; reader 3: 87.5%) at PB MRI and 75.0% (reader 2: 75.0%; reader 3: 100%) at follow-up examination for cases with LR.

### Interreader agreement

The consistency among readers ranged between slight, fair, and moderate agreement: For presence of a mass, *κ* was 0.34; for signal changes on T2-weighted imaging, 0.08; for signal changes on T1-weighted imaging, 0.2; for evaluation of contrast enhancement, 0.13; and for signal changes in ADC, 0.55.

## Discussion

This study investigated the impact of a PB MRI on the diagnostic confidence and performance in detecting LR of STS of the limb. Eight out of 72 patients after multimodal therapy showed histologically proven locally recurrent STS. The most important finding of this retrospective analysis was that the presence of a PB MRI improves diagnostic confidence in detecting LR of STS of the limb. Furthermore, when looking at the diagnostic performance, specificity slightly increased; however, not to a significant level. Evaluating standard MRI parameters after multimodal therapy for STS of the limb over time, we saw that in both groups most imaging characteristics remained predominantly constant between the PB MRI at 6 months after surgery and at approximately 36 months at time of LR. Although this time interval deviates from the recommendations of the ESMO guidelines for early routine follow-up, it demonstrates that imaging characteristics do not change relevantly over a longer period of time. This is relevant for the interpretation of late follow-up, including the case where some preliminary examinations are not available. Further, presence of a mass showed highest diagnostic performance of all assessed imaging parameters in detection of LR of STS.

While the performance of MRI in diagnosing LR of STS has been examined in several previous studies [16, 20–29] and has recently been brought together in a systematic review [18], the availability of a PB MRI on diagnostic confidence and performance in detecting LR in multimodally treated STS of the limb has not been investigated yet. Dealing with this subject, our study revealed that the availability of a baseline MRI scan after primary treatment of STS of the limb improves diagnostic confidence in detecting LR.

Analogous to previous studies [18], the presence of a mass appears a useful diagnostic MRI criterion to diagnose LR of STS as it showed highest diagnostic performance with an AUC varying between 73.1 and 83.6% for the three readers in our study with significant differences between the patient group with and without LR. A recent systematic review reported fairly high sensitivity and specificity with pooled values of 80.9% and 77.0%, respectively [18]. In contrast, signal characteristics at standard T2- and T1-weighted imaging did not show significant differences (despite signal changes on T1-weighted images for reader 3) between the groups with and without LR, and therefore do not substantially contribute to the detection of LR in STS. This observation is in line with current literature as signal characteristics on both T1- and T2-weighted imaging lacked specificity [18], which might particularly be due to variable severity and duration of radiation-induced soft-tissue signal intensity changes [30, 31]. Similarly, the use of contrast-enhanced fat saturated T1-weighted images revealed no significant benefit for the detection of LR in STS with low AUCs between 52.4 and 62.5% for the readers in our study. The diagnostic performance of MRI with and without the use of gadolinium-based contrast agents has not been conclusively clarified yet. Some previous studies reported that both sensitivity and specificity were not significantly different regardless of readers’ experience for contrast-enhanced and non-enhanced MRI analysis for locally recurrent STS [21, 23], while in contrast another study showed that the use of gadolinium significantly improved sensitivity [32]. It becomes apparent that further research is needed to clarify this question, in particular, when patients have contraindications for gadolinium-based contrast agents and are in need of imaging follow-up after multimodally treated STS. A small part of patients in our study (*n* = 35) received additional DWI with ADC mapping, showing significant differences between both groups with LR and without LR for all readers with AUCs varying between 65.6 and 96.6%. Based on current literature, functional MRI techniques including DWI and quantitative dynamic contrast-enhanced (DCE)-MRI seem to add value, making the diagnosis of LR in STS [16, 18]. However, we did not analyze the benefit of DCE-MRI in the present study. Rather, we have investigated the development and progression of imaging parameters over time (6–36 months after multimodal therapy of STS and at time of LR) and were able to show that most imaging characteristics remained predominantly constant over time in both patients with as well as without LR of STS. This is an important finding and has to be taken into account in the post-treatment course of STS as routine MRI follow-up is commonly performed at regular intervals [4, 11] and signal alterations, e.g., streaked or circumscribed T2-hyperintensities, may either be caused by post-therapeutic changes or may also be caused by LR.

Another interesting fact is that the interreader agreement regarding the imaging characteristics was quite poor (*ĸ* 0.08–0.55). However, some previous studies did not calculate interreader agreement, e.g., when reading was done in consensus [26]. In contrast, in a previous study of Del Grande, interreader agreement was very high (*ĸ* 0.84–1.0) [33] however calculated with only two readers. The different results might be explained by the fact that generally robust estimation of interrater agreement is dependent on the number of readers [34]. Therefore, a limitation of the best performing parameter “presence of a mass” is the only fair interreader agreement.

We acknowledge several limitations to our study. First, the number of patients with LR was relatively small. With an amount of 11%, this is even less than reported in the literature with an estimated 16% of tumor recurrence after a 24-month follow-up [17, 35]. An explanation might be that patients in our institution received regional hyperthermia as additional treatment option and secondly, of course, there is a risk of selection bias.

Second, only a part of the patients included in this study underwent DWI with ADC mapping, which might have impaired our results. Nevertheless, the results with regard to DWI are in line with previous studies. Furthermore, STS contain a broad spectrum of variable signal intensity depending on the histological tumor entity. For example, myxoid tumors are well known for mimicking benign cysts in non-enhanced MRI exams. However, a further subgroup analysis was not feasible due to the limited number of patients with LR in the present study. Another limitation is the fact that although mean time interval for the second examination was similar between both cohorts, the standard deviation was distinct higher in the group of LR. This is because we did not want to rule out an outlier, who presented with LR approximately 9 years after multimodal therapy, due to the low number of recurrences. However, in both cohorts the mean time interval was approximately 36 months. Another limitation is that the study was not designed and is not able to provide information on whether the follow-up interval needs to be changed. Among other things, this depends on further clinical parameters and examination methods; for example, the rate and time interval of local recurrence of STS.

## Conclusion

In conclusion, the availability of a PB MRI improves diagnostic confidence in detecting LR of STS of the limb. Further, the presence of a PB MRI slightly increased specificity, however, not to a significant level. According to current knowledge, the presence of a mass seems to be the most reliable MRI parameter in detecting LR in STS.

## Data Availability

The data that support the findings of this study are available on reasonable request from the corresponding author, [SSG]. The data are not publicly available due to containing information that could compromise the privacy of research participants.
